# Comparative study of stretched-exponential and kurtosis models of diffusion-weighted imaging in renal assessment to distinguish patients with primary aldosteronism from healthy controls

**DOI:** 10.1371/journal.pone.0298207

**Published:** 2024-02-08

**Authors:** Deying Wen, Pengfei Peng, Xun Yue, Chenxiao Xu, Qian Pu, Yue Ming, Huiyi Yang, Miaoqi Zhang, Yan Ren, Jiayu Sun

**Affiliations:** 1 Department of Radiology, West China Hospital, Sichuan University, Chengdu, China; 2 Department of Radiology, Affiliated Hospital of North Sichuan Medical College, Nanchong, China; 3 Department of Endocrinology and Metabolism, West China Hospital, Sichuan University, Chengdu, China; 4 GE Healthcare, MR Research, Beijing, China; Memorial Sloan Kettering Cancer Center, UNITED STATES

## Abstract

**Purpose:**

To compare the ability of diffusion parameters obtained by stretched-exponential and kurtosis models of diffusion-weighted imaging (DWI) to distinguish between patients with primary aldosteronism (PA) and healthy controls (HCs) in renal assessment.

**Materials and methods:**

A total of 44 participants (22 patients and 22 HCs) underwent renal MRI with an 11 b-value DWI sequence and a 3 b-value diffusion kurtosis imaging (DKI) sequence from June 2021 to April 2022. Binary logistic regression was used to construct regression models combining different diffusion parameters. Receiver-operating characteristic (ROC) curve analysis and comparisons were used to evaluate the ability of single diffusion parameters and combined diffusion models to distinguish between the two groups.

**Results:**

A total of six diffusion parameters (including the cortical anomalous exponent term [α_Cortex], medullary fractional anisotropy [FA_Medulla], cortical FA [FA_Cortex], cortical axial diffusivity [Da_Cortex], medullary mean diffusivity [MD_Medulla] and medullary radial diffusivity [Dr_Medulla]) were included, and 10 regression models were studied. The area under the curve (AUC) of Dr_Medulla was 0.855, comparable to that of FA_Cortex and FA_Medulla and significantly higher than that of α_Cortex, Da_Cortex and MD_Medulla. The AUC of the Model_all parameters was 0.967, comparable to that of Model_FA (0.946) and Model_DKI (0.966) and significantly higher than that of the other models. The sensitivity and specificity of Model_all parameters were 87.2% and 95%, respectively.

**Conclusion:**

The Model_all parameters, Model_FA and Model_DKI were valid for differentiating between PA patients and HCs with similar differentiation efficacy and were superior to single diffusion parameters and other models.

## Introduction

Primary aldosteronism is a major cause of secondary hypertension. The prevalence of PA is approximately 5–20% in patients with hypertension and 8.9–33% in patients with refractory hypertension [1, 2]. It is characterized by the autonomous secretion of excess aldosterone from the adrenal cortex, independent of renin, angiotensin II and sodium status. The resulting overactivation of mineralocorticoid receptors leads to detrimental effects such as volume expansion, hypertension, hypokalemia, metabolic alkalosis, and an increased risk of cardiovascular and renal diseases [3]. Numerous studies have demonstrated that prolonged exposure to high aldosterone levels can cause damage to the kidneys through a variety of mechanisms [4–6]. The risk of developing chronic kidney disease is 4–12 times greater in patients with PA than in patients with essential hypertension [7], and approximately 11.7% of patients with PA already have chronic kidney disease at the time of initial diagnosis [8]. In clinical practice, the estimated glomerular filtration rate (eGFR) is often used as a biological indicator for renal evaluation. However, the eGFR may not be highly accurate in patients with PA due to potential glomerular hyperfiltration, leading to exaggerated results [9, 10]. The eGFR may be within the normal range, but the kidney is actually damaged. Therefore, early and precise evaluation of renal injury is crucial for implementing effective renal protection strategies and approaches during PA treatment.

Diffusion kurtosis imaging (DKI), as a new diffusion model, utilizes a higher b-value to characterize the non-Gaussian diffusive motion of water molecules in biological tissues, quantifies the size and direction of water molecule diffusion, and allows a more accurate assessment of the complexity of tissue microstructures [11–14]. DKI was first described in studies in 2004 and 2005 [15] and was initially used exclusively for brain imaging [16, 17]. In 2014, a study first reported the feasibility of DKI in the kidneys of healthy volunteers [18]. Over the past decade, numerous studies have investigated the application of DKI in kidney diseases to assess renal injury, such as renal tumors [19–21], acute kidney injury from various causes [12, 22, 23], chronic kidney disease [13, 24, 25], diabetic nephropathy [12, 26, 27], IgA nephropathy [28, 29], and lupus nephritis [30]. However, few studies have applied DKI to assess renal injury in patients with PA.

The stretched-exponential model is another model that describes the non-Gaussian behavior of water molecule diffusion and can provide some information for quantifying tissue heterogeneity [30, 31]. Several studies have reported that lower α values indicate a more heterogeneous environment [31, 32]. Its application in the kidney mostly involves tumors and lupus nephritis and rarely in PA.

Our previous study [33] reported significant differences in certain DKI-derived parameters (including FA and Da in the cortex; FA, MD and Dr in the medulla) and a stretched-exponential model-derived parameter (cortical α) between PA patients and healthy control participants. To our knowledge, this is the first study to evaluate the kidneys of PA patients using the stretched-exponential and kurtosis models of DWI. The purpose of the previous study was to compare whether there were differences in each diffusion parameter of various DWI models between patients with PA and healthy volunteers and to analyze the correlation between diffusion parameters and clinical indicators in PA patients. In total, we examined 13 diffusion parameters in 4 DWI models and ultimately found that 6 diffusion parameters were significantly different between patients and healthy volunteers. Additionally, plasma aldosterone and the eGFR were correlated with medullary FA in PA patients. However, the efficacy of these diffusion parameters for renal assessment has not been explored in depth. How well do these six diffusion parameters from different diffusion models (DKI and stretched DWI) and different renal structures (cortex and medulla) distinguish between PA patients and healthy volunteers? Is it possible to simplify the diffusion parameters with different regression combinations to efficiently differentiate between the two? Could it even be simplified to the point where medullary FA can efficiently differentiate between PA patients and healthy volunteers? Therefore, the aim of this study was to quantitatively compare the ability of these diffusion parameters to assess the kidneys of PA patients and to distinguish between patients with PA and healthy volunteers.

## Materials and methods

### Study population

This study was approved by the local biomedical ethics committee (No. 2019 145), and written informed consent was obtained from each participant before the MRI examination.

A total of 44 participants were enrolled in the study from June 2021 to April 2022; 22 PA patients with a mean age of 48±10 years and 22 healthy volunteers with a mean age of 45±11 years were included. The specific inclusion criteria for patients were as follows: (1) diagnosed with PA according to the American Endocrine Society Clinical Practice Guideline in 2016 [34]: (i) had spontaneous hypokalemia, a plasma renin concentration below the limit of detection, and a plasma aldosterone concentration (PAC) > 20 ng/dL; (ii) had a positive plasma aldosterone-to-renin ratio (ARR) and one or more positive confirmatory tests (captopril challenge test and saline infusion test); (2) had no specific treatment (adrenalectomy or mineralocorticoid receptor antagonist) after diagnosis of PA; (3) aged ≥ 18 years; and (4) had no other kidney diseases or diseases affecting the kidney. The specific inclusion criteria for healthy volunteers were as follows: had (1) no known acute or chronic disease, (2) no kidney disease or disease affecting the kidney, and (3) no medication that affects the physiological function of the kidney within the last half month. The exclusion criteria for all participants were as follows: (1) had poor-quality MR images that could not be analyzed and (2) had contraindications to MR examinations, claustrophobia or other psychiatric disorders that prevented them from cooperating with MR examinations.

### Image analysis

**Fig 1:**
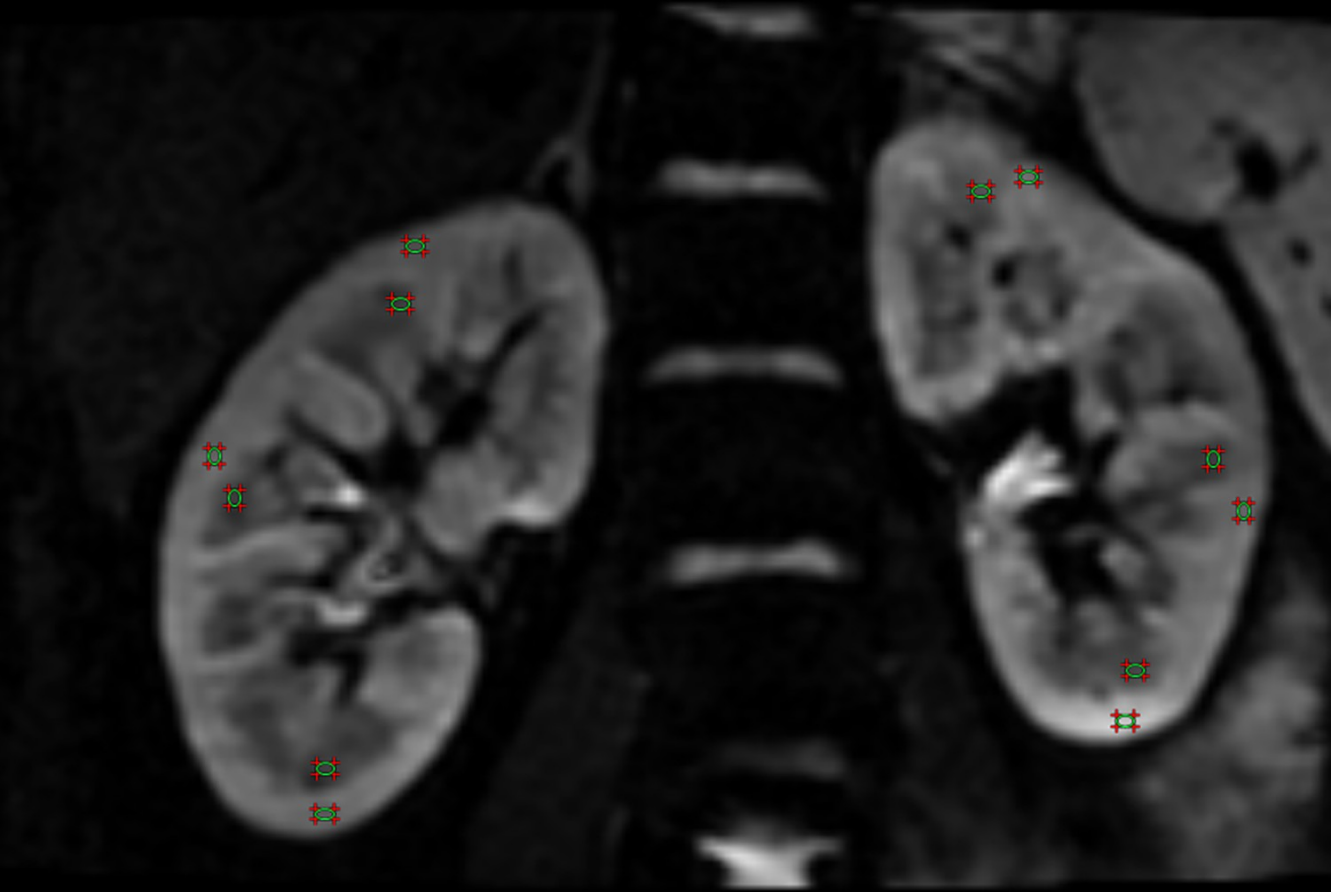
The placement of ROIs. The ROIs were placed both in the cortex and medulla of the three regions of the kidney (upper pole, real hilum and lower pole) on the b = 0 s/mm^2^ DW images.

The relevant diffusion parameters of the stretched-exponential model and DKI were measured separately using the region of interest (ROI) technique as reported in previous study [33]. The placement of the ROIs is shown in Fig 1. All the image measurements were performed by two independent observers who had no knowledge of the clinical diagnosis.

### Regression models for differentiating effectiveness

Based on a previous study [33], the following six parameters that differed significantly between PA patients and healthy individuals were selected to construct the model: α, FA and Da in the cortex (α_Cortex, FA_Cortex and Da_Cortex) and FA, MD and Dr in the medulla (FA_Medulla, MD_Medulla and Dr_Medulla). (1) Five models based on the classification of the nature of the diffusion parameters were developed: Model_α, Model_FA (including FA_Cortex and FA_Medulla), Model_D (including Da_Cortex, MD_Medulla and Dr_Medulla), Model_DKI (including FA_Cortex, Da_Cortex, FA_Medulla, MD_Medulla and Dr_Medulla) and Model_all parameters (including all six parameters). (2) Five models were constructed by classifying the renal parenchyma: Model_DKI_Cortex (including FA_Cortex and Da_Cortex); Model_DKI_Medulla (including FA_Medulla, MD_Medulla and Dr_Medulla); Model_DKI (including FA_Cortex, Da_Cortex, FA_Medulla, MD_Medulla and Dr_Medulla); Model_Cortex (including α_Cortex, FA_Cortex and Da_Cortex); and Model_Medulla (including FA_Medulla, MD_Medulla and Dr_Medulla).

### Statistical analysis

The data are expressed as the mean ± standard deviation or median and interquartile range. The ability of the six diffusion parameters to distinguish PA patients from healthy control participants was calculated using ROC curves; then, binary logistic regression was used to construct regression models combining different diffusion parameters to distinguish PA patients from healthy controls, and a total of 10 regression models were constructed. ROC curves were compared for different parameters and regression models, and the area under the ROC curve was calculated. The sensitivity and specificity were also calculated by the Youden index. We performed the statistical analysis using SPSS 25.0 (IBM, Corp.) and MedCalc (version 20.218) statistical software. *p* < 0.05 was considered to indicate statistical significance.

**Fig 2:**
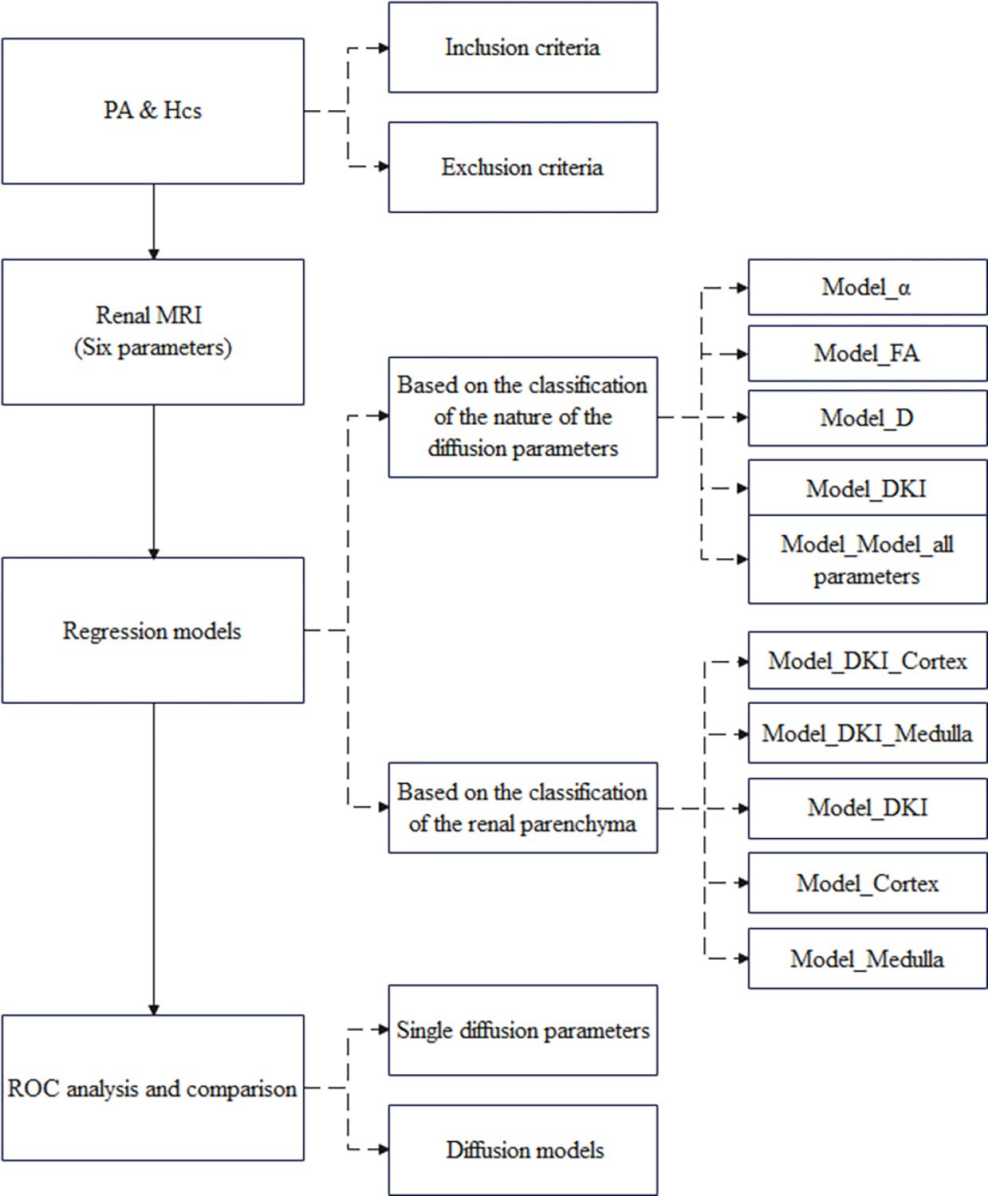
Study flow diagram.

The study steps are presented in a flow diagram (Fig 2).

## Results

### Six diffusion parameters

The interobserver agreement between the two observers, assessed by intraclass correlation coefficient (ICC) ranged from good to excellent.

**Fig 3:**
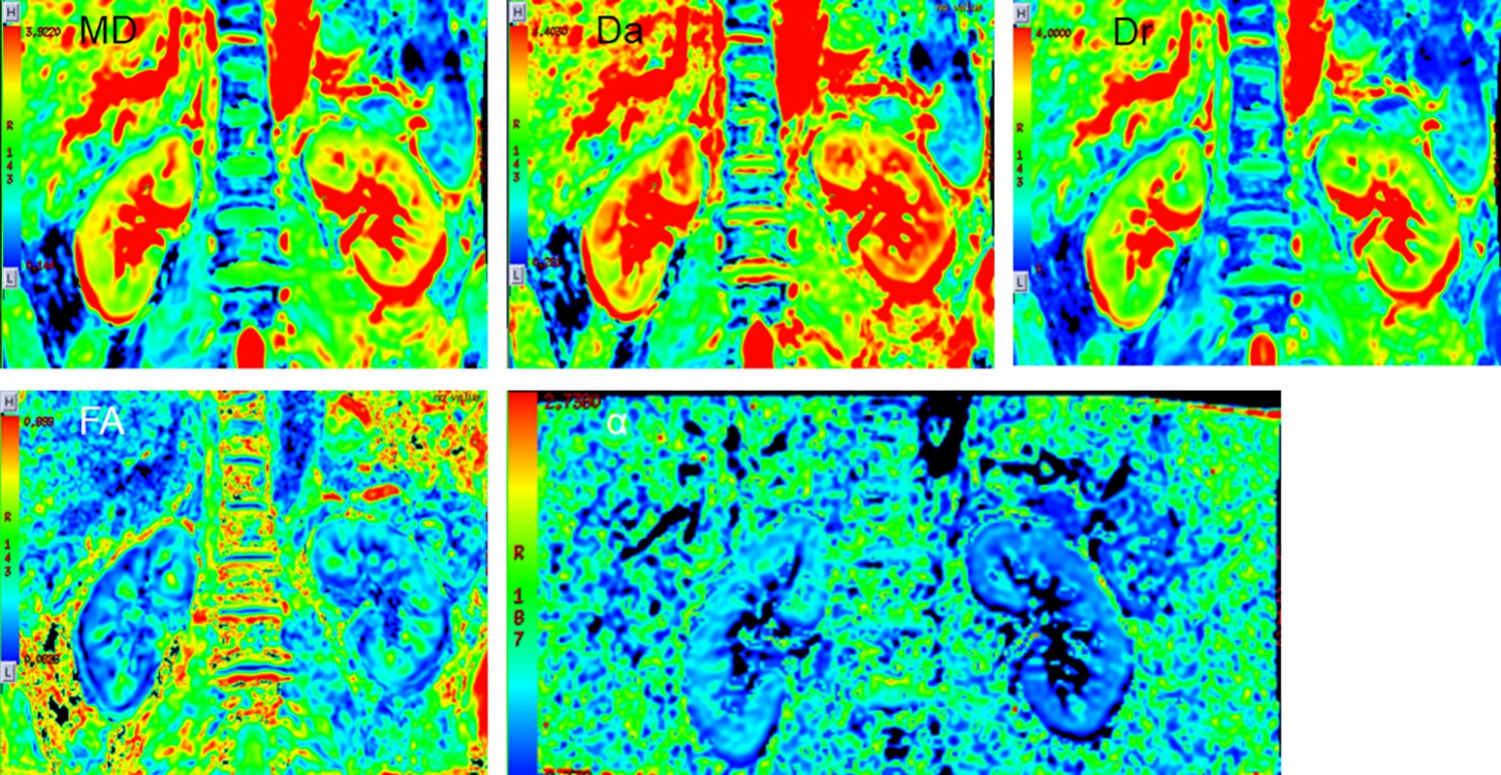
Typical functional maps of the six parameters for PA patients.

The results of the six parameters are shown in Table 1, and the typical functional maps of the six parameters for PA patients is shown in Fig 3.

**Table 1:** Comparison of six diffusion parameters between patients with PA and HCs.

Parameters	α*_Cortex	FA*_Cortex	Da_Cortex	FA*_Medulla	MD_Medulla	Dr_Medulla
PA Patients	752.319±102.912	0.214(0.195–0.272)	3.590(3.320–3.933)	0.301(0.266–0.383)	2.679±0.449	2.220±0.234
HCs	704.121±79.963	0.296(0.270–0.339)	4.003(3.605–4.356)	0.231(0.208–0.276)	2.981±0.304	2.583±0.254
*p* value	0.016	<0.001	0.001	<0.001	<0.001	<0.001

**Notes:** Normally distributed data are expressed as the mean ± standard deviation and nonnormally distributed data are expressed as the median and interquartile range.

HCs, healthy controls; FA, fractional anisotropy; Da, axial diffusivity; MD, mean diffusivity; Dr, radial diffusivity.

*FA and α have no units, and the units of the Da, MD, and Dr values are * 10^−3^ mm^2^/s.

### ROC curve comparison for single diffusion parameters

**Fig 4:**
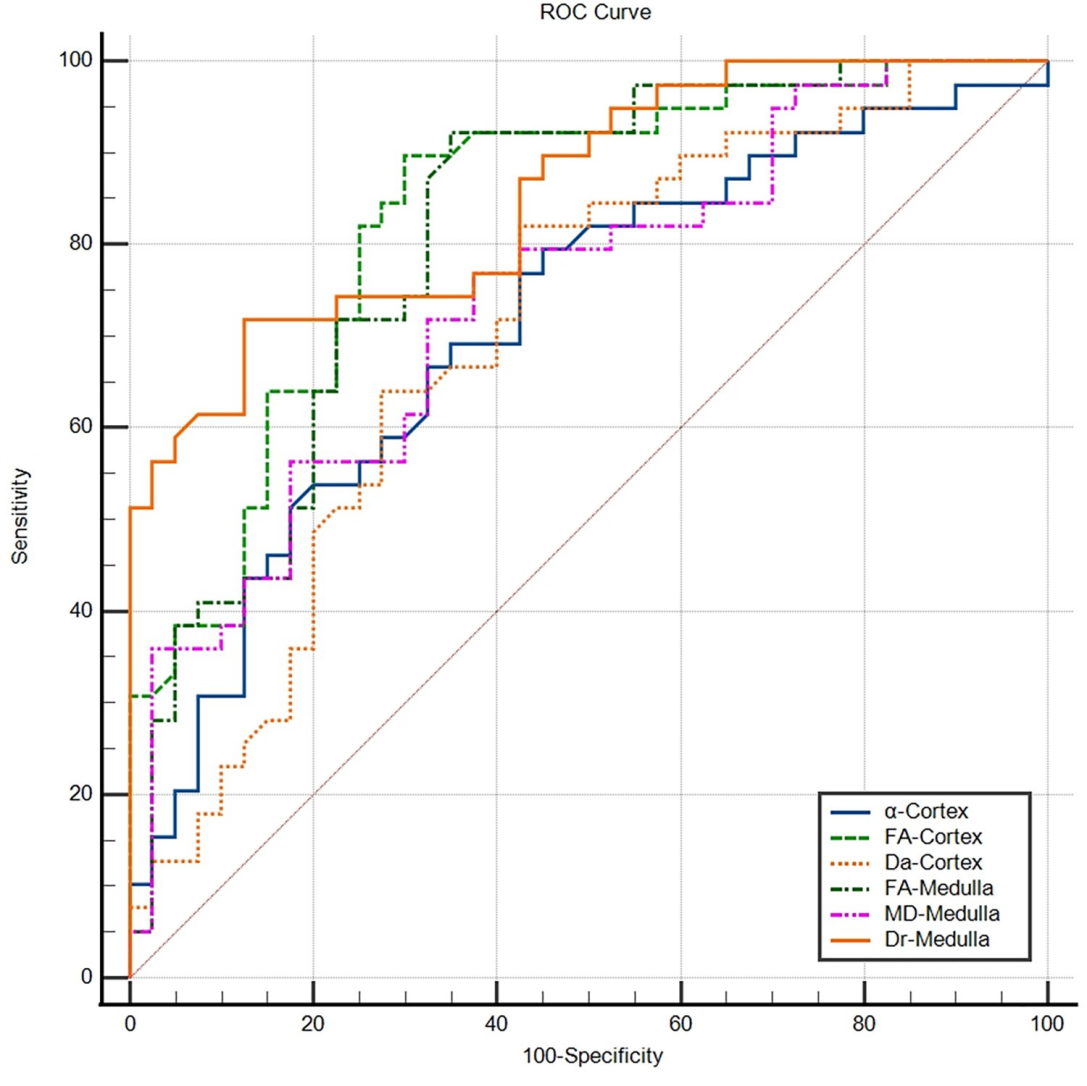
ROC curves of the single diffusion parameters for distinguishing patients with PA from HCs. The AUC values for the α_Cortex, FA_Cortex, Da_Cortex, FA_Medulla and MD_Medulla were 0.715, 0.834, 0.708, 0.813, 0.741 and 0.855, respectively.

The AUC for Dr_Medulla was 0.855, which was the highest among all the parameters and was significantly greater than that for α_Cortex, Da_Cortex and MD_Medulla; moreover, no statistically significant differences were found for FA_Cortex or FA_Medulla. However, the sensitivity of Dr_Medulla was only 87.5%, which was lower than that of FA_Medulla (90%), and the specificity was only 72.50%, which was lower than that of FA_Cortex (89.7%). The results of the ROC curve analyses of the different diffusion parameters are presented in Tables 2 and 3 and Fig 4.

**Table 2:** ROC curve results for six diffusion parameters.

Variable	AUC	95% CI	Youdenindex	Cutoff value(x10-3 mm2/sec)	Sensitivity	Specificity
α*_Cortex	0.715	0.602–0.811	0.345	711.333	79.50%	55%
FA*_Cortex	0.834	0.733–0.908	0.597	0.281	70%	89.70%
Da_Cortex	0.708	0.595–0.805	0.396	3.985	57.50%	82.10%
FA*_Medulla	0.813	0.709–0.892	0.55	0.241	90%	65%
MD_Medulla	0.741	0.630–0.833	0.4	2.707	82.50%	57.50%
Dr_Medulla	0.855	0.758–0.924	0.6	2.325	87.50%	72.50%

**Notes:** FA, fractional anisotropy; Da, axial diffusivity; MD, mean diffusivity; Dr, radial diffusivity; AUC, area under curve; 95% CI, 95% confidence interval.

*FA and α have no units.

**Table 3:** Results of ROC curve comparisons for different diffusion parameters.

Variable	FA_Cortex	Da_Cortex	FA_Medulla	MD_Medulla	Dr_Medulla
α_Cortex	0.0881	0.9314	0.2361	0. 7236	0.0395
FA_Cortex		0 .0206	0. 7758	0.1446	0.7290
Da_Cortex			0.2059	0.5383	0.0075
FA_Medulla				0.3686	0.4468
MD_Medulla					0.0003

**Notes:** The AUC of Dr_Medulla was significantly greater than those of α_Cortex, Da_Cortex, and MD_Medulla, and no statistically significant differences were found between FA_Cortex and FA_Medulla.

### ROC curve comparison for different diffusion models

**Fig 5:**
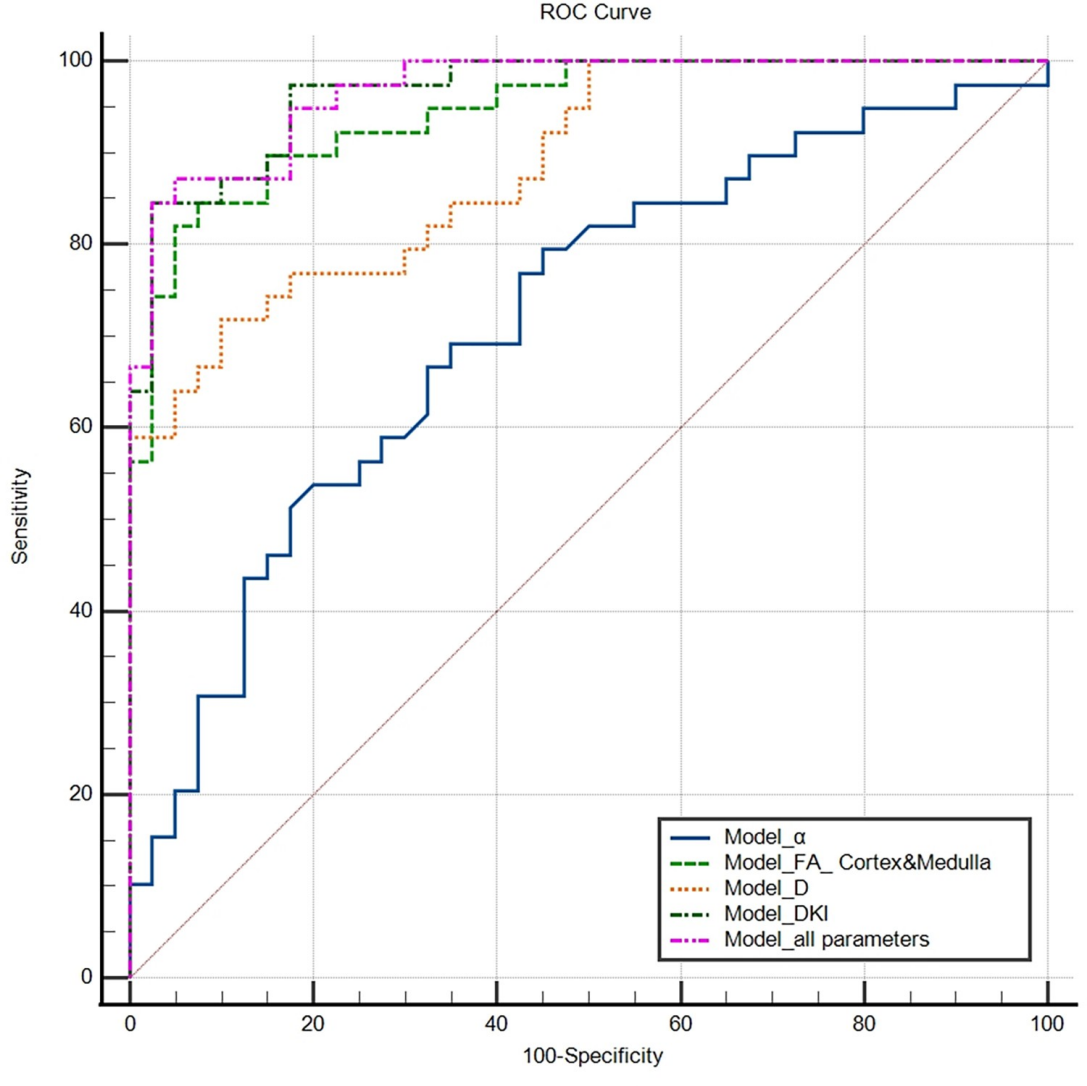
ROC curves of the different diffusion models for distinguishing patients with PA from HCs. The AUC values of the Model_α, Model_FA, Model_D, Model_DKI and Model_all parameters were 0.715, 0.946, 0.885, 0.813, 0.966 and 0.967, respectively.

The AUC of the Model_all parameters was 0.967, which was the highest among all the models and was significantly greater than that of Model_α and Model_D; moreover, no statistically significant differences were found between Model_FA and Model_DKI. The highest sensitivity was obtained with Model_all parameters (87.20%), and the highest specificity was obtained with Model_DKI (97.5%). The results of the ROC curve comparison of the different models are displayed in Tables 4 and 5 and Fig 5.

**Table 4:** ROC curve results for different diffusion models.

Variable	AUC	95% CI	Youden index	Sensitivity	Specificity
Model_α	0.715	0.602–0.811	0.345	79.50%	55%
Model_FA	0.946	0.870–0.984	0.771	84.60%	92.50%
Model_D	0.885	0.794–0.946	0.618	71.80%	90%
Model_DKI	0.966	0.899–0.994	0.821	84.60%	97.50%
Model_all parameters	0.967	0.901–0.994	0.822	87.20%	95%

**Notes:** Model_FA contains the parameters FA_Cortex and FA_Medulla. Model_D contains the parameters Da_Cortex, MD_Medulla and Dr_Medulla. Model_DKI contains the parameters FA_Cortex, Da_Cortex, FA_Medulla, MD_Medulla and Dr_Medulla. Model_all parameters contains all six diffusion parameters.

**Table 5:** Results of ROC curve comparisons for different diffusion models.

Variable	Model_FA	Model_D	Model_DKI	Model_all parameters
Model_α	0.0002	0.0127	<0.0001	<0.0001
Model_FA		0.0494	0.2111	0.1761
Model_D			0.0052	0.0050
Model_DKI				0.6724

**Notes:** The AUC values of Model_FA, Model_DKI and Model_all parameters were significantly greater than those of Model_α and Model_D, and no statistically significant differences were found among the three models.

### ROC curve comparison for different renal parenchymal models

**Fig 6:**
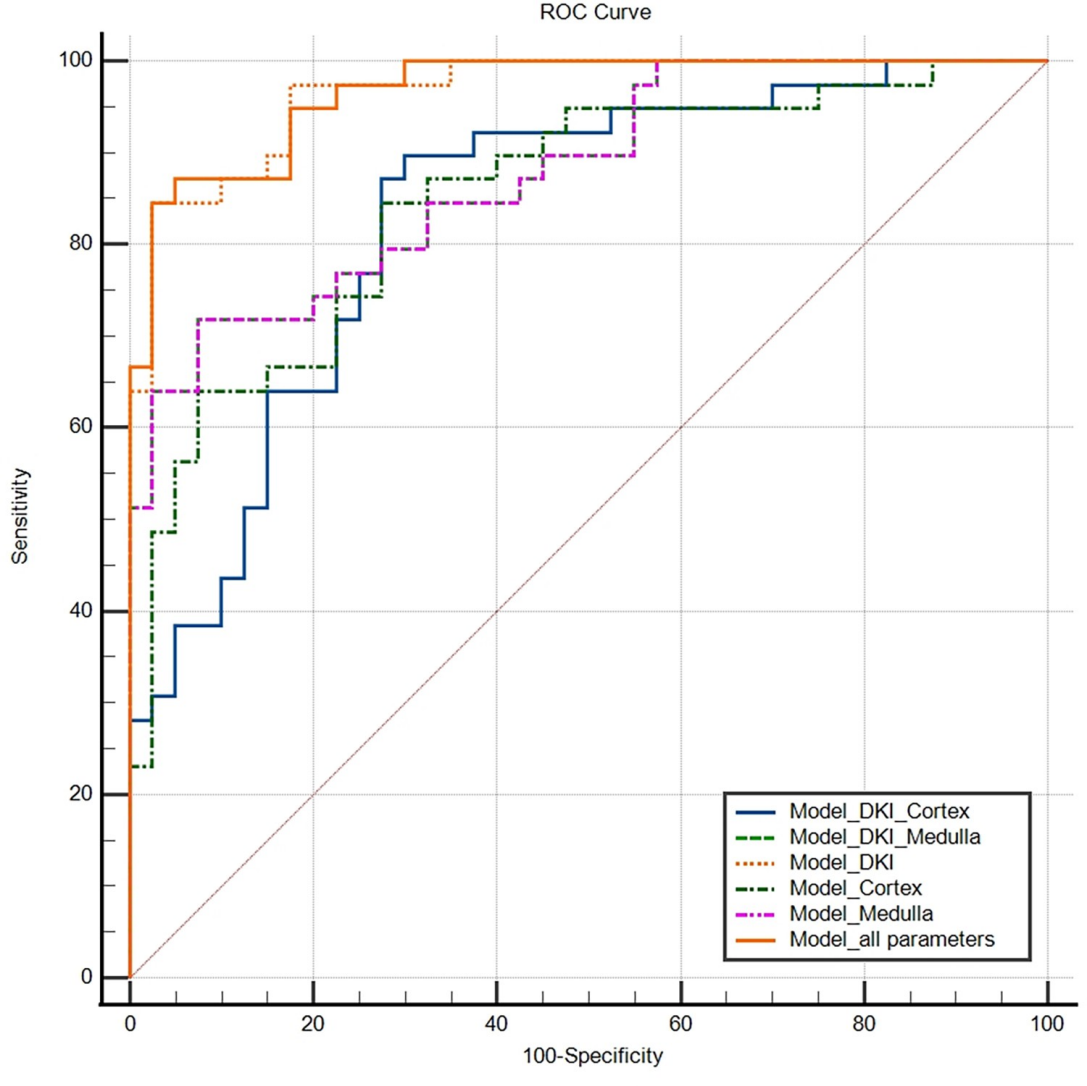
ROC curves of the different renal parenchymal models for distinguishing patients with PA from HCs. The AUC values of the Model_DKI_Cortex, Model_DKI_Medulla, Model_DKI, Model_Cortex, Model_Medulla and Model_all parameters were 0.833, 0.877, 0.966, 0.851,0.877 and 0.967, respectively.

The AUC values of the Model_DKI_Cortex, Model_DKI_Medulla, Model_DKI, Model_Cortex, Model_Medulla and Model_all parameters were 0.833, 0.877,0.966, 0.851, 0.877 and 0.967, respectively. The AUC values of the of Model_DKI and Model_all parameters were comparable and significantly greater than those of the other 4 models. The sensitivity and specificity of Model_DKI were 84.6% and 97.5%, respectively. The sensitivity and specificity of Model_all parameters were 87.2% and 95%, respectively. The results of the ROC curve comparison are presented in Tables 6 and 7 and Fig 6.

**Table 6:** ROC curve results of different renal parenchymal models.

Variable	AUC	95% CI	Youden index	Sensitivity	Specificity
Model_DKI_Cortex	0.833	0.733–0.908	0.597	89.70%	70%
Model_DKI_Medulla	0.877	0.784–0.940	0.643	71.80%	92.50%
Model_DKI	0.966	0.899–0.994	0.821	84.60%	97.50%
Model_Cortex	0.851	0.753–0.921	0. 571	84.60%	72.50%
Model_Medulla	0.877	0.784–0.940	0.643	71.80%	92.50%
Model_all parameters	0.967	0.901–0.994	0.822	87.20%	95%

**Notes:** Model_DKI_Cortex contains the parameters FA_Cortex and Da_Cortex. Model_DKI_Medulla contains the parameters FA_Medulla, MD_Medulla and Dr_Medulla. Model_DKI contains the five diffusion parameters except for α. Model_Cortex contains the parameters α_Cortex, FA_Cortex and Da_Cortex. Model_Medulla contains the parameters FA_Medulla, MD_Medulla and Dr_Medulla.

**Table 7:** Results of ROC curve comparisons for different renal parenchymal models.

Variable	Model_DKI_Medulla	Model_DKI	Model_Cortex	Model_Medulla	Model_all parameters
Model_DKI_Cortex	0.4842	0.0015	0.4606	0.4842	0.0014
Model_DKI_Medulla		0.0059	0.6515	1.000	0.0058
Model_DKI			0.0034	0.0059	0.6724
Model_Cortex				0.6515	0.0024
Model_Medulla					0.0058

**Notes:** The AUC values of the Model_DKI and Model_all parameters were significantly greater than those of the other models, and no statistically significant differences were found between the two models.

## Discussion

In this study, we compared the ROC curves of individual parameters and different models to identify the parameter or model with the best diagnostic accuracy for differentiating between patients with PA and HCs. Our findings revealed that the distinguishing ability of Model_all parameters (AUC, 0.967; sensitivity, 87.2%; specificity, 95%) was comparable to that of Model_FA (AUC, 0.946; sensitivity, 84.6%; specificity, 92.5%) and Model_DKI (AUC, 0.966; sensitivity, 84.6%; specificity, 97.5%) and was significantly higher than that of other models and single diffusion parameters. On the other hand, the stretched exponential model demonstrated a much weaker differentiation efficiency (AUC = 0.715).

DWI is a functional magnetic resonance imaging method that can noninvasively measure the diffusive motion of water molecules in vivo and involves numerous diffusion models; the stretched-exponential model and the diffusion kurtosis model are two of the many models that can describe the non-Gaussian behavior of water. Both models require sequences with multiple b-values, and in our study, the stretched-exponential model employed a total of 11 b-values: 0, 10, 30, 50, 100, 150, 200, 400, 600, 800 and 1000 s/mm^2^; the DKI employed 3 b-values: 0, 500, and 1000 s/mm^2^. Respiratory gating in the free-breathing state was used to acquire coronal images, and the total time was slightly longer, depending on the patient’s respiratory rate; approximately 7 minutes or more for the stretched-exponential model and approximately 5 minutes or more for DKI.

The stretched-exponential model of DWI derived two diffusion parameters (distributed diffusion coefficient [DCC] and α) with an 11 b-value DWI sequence. α is usually thought to reflect tissue heterogeneity and is used to assess tumors. Previous studies have suggested that lower α values indicate higher tissue heterogeneity [31, 35, 36]. Furthermore, a study reported that the activity index was correlated with the medullary α, and it had superior performance in differentiating “proliferative” lupus nephritis [30]. The mechanism of kidney injury in patients with PA is complex and unclear and may differ from that in patients with tumors and lupus nephritis. A previous study reported that there was a degree of interstitial fibrosis and inflammatory cell infiltration in the kidneys of PA patients [6], which may have led to reduced heterogeneity. On the other hand, the excessive secretion of aldosterone leads to swelling and dysfunction of endothelial cells, which stiffens blood vessels and increases peripheral resistance, thus leading to a decrease in renal blood flow [6]. A decrease in blood flow is captured by α in the cortex and consequently increases. This may be similar to the interpretation of the results of a previous study on nonalcoholic fatty liver disease (NAFLD) in rabbits, where they found that α increased with increasing severity of NAFLD because of the decrease in blood flow [37]. Nevertheless, the exact physiological basis of the α remains unclear, necessitating further in-depth studies to establish a more reliable explanation. Moreover, the differentiation efficiency of the stretched-exponential model (AUC = 0.715) was much lower than that of the DKI model (AUC = 0.966).

In the present study, Dr_Medulla exhibited the highest differentiation efficiency (AUC = 0.855) among the individual diffusion parameters and was comparable to that of medullary FA and cortical FA. DKI, as an extension of DTI, is thought to have the potential to better reflect the complexity of the microstructure of biological tissues and may be more accurate and sensitive in assessing the microstructure of the kidney [24]. The diffusivity parameters obtained by DKI, including mean diffusivity (MD), radial diffusivity (Dr) and axial diffusivity (Da), represent non-Gaussian distributed diffusion coefficients and depend mainly on the diffusion of water molecules in tissue; these parameters are limited when pathological changes result in high cell density and narrow extracellular spaces [38]. Renal vascular fibrosis caused by elevated blood pressure, endothelial cell swelling and dysfunction caused by excess aldosterone, and segmental glomerulosclerosis may all contribute to greater sensitivity of Dr_Medulla. FA reflects the amount of anisotropy, and the kidney is anatomically characterized by a radial arrangement of renal tubules, collecting systems and blood vessels from the medulla to the cortex [39]; therefore, alterations in the microstructure of these radially arranged tubules, collecting systems and blood vessels in the kidney may lead to changes in FA values. Glomerulosclerosis, vascular stiffness and tubular damage in patients with PA may be responsible for the greater sensitivity of FA.

In addition to comparing individual parameters, ROC curves were compared among the different models to determine which model had the best differentiation accuracy. Our study demonstrated that among all the combined models, Model_FA, which included only two parameters (medullary FA and cortical FA), had a comparable advantage to Model_DKI and Model_all parameters for differentiating between patients and control participants. That is, the inclusion of diffusivity parameters (including MD_Medulla, Dr_Medulla and Da_Cortex) and α did not increase the discriminatory efficacy of the models, and the combination of cortical FA with medullary FA showed good diagnostic performance, much greater than that of FA alone or any of the individual diffusion parameters. However, Model_FA was not the most sensitive or specific, with a lower sensitivity than Model_all parameters and a lower specificity than Model_DKI. Therefore, we propose to rationalize the use of different combinations of parameters by comprehensively evaluating the discriminatory ability, specificity, sensitivity, and sequence acquisition of parameters to efficiently differentiate PA patients from healthy volunteers.

There were several limitations in our study that should be acknowledged. First, the patient population was relatively small because of the strict inclusion (age ≥ 18 years, confirmed diagnosis of PA with no previous treatment, and no kidney disease) and exclusion criteria. In addition, the nature of the hospital makes it challenging to recruit sufficient patients who meet the criteria because most of the patients had been previously treated elsewhere. Therefore, additional effort and time are needed to expand the patient population for further validation. Second, this was a single-center study. Third, in this study, we used the manual ROI method, and some studies recommend the use of semiautomated segmentation techniques; however, Li LP et al. [40] compared the manual ROI method with semiautomated segmentation techniques in the assessment of chronic kidney disease using BOLD MR and found a high degree of agreement between the two techniques in R2* measurements. Therefore, we will use semiautomated segmentation techniques for further assessment in subsequent studies and compare the agreement of the two techniques.

In conclusion, our study demonstrated that Model_all parameters, Model_FA, and Model_DKI were effective at differentiating between PA patients and healthy volunteers, with similar efficacy. These models outperformed single diffusion parameters and other models in terms of discrimination.

## References

[1] Jaffe G, Gray Z, Krishnan G, Stedman M, Zheng Y, Han J, et al. Screening Rates for Primary Aldosteronism in Resistant Hypertension: A Cohort Study. Hypertension. 2020;75(3):650–659. doi: 10.1161/HYPERTENSIONAHA.119.14359 32008436

[2] Lin X, Ullah MHE, Wu X, Xu F, Shan SK, Lei LM, et al. Cerebro-Cardiovascular Risk, Target Organ Damage, and Treatment Outcomes in Primary Aldosteronism. Front Cardiovasc Med. 2022;8:798364. Published 2022 Feb 2. doi: 10.3389/fcvm.2021.798364 35187110 PMC8847442

[3] Vaidya A, Mulatero P, Baudrand R, Adler GK. The Expanding Spectrum of Primary Aldosteronism: Implications for Diagnosis, Pathogenesis, and Treatment. Endocr Rev. 2018;39(6):1057–1088. doi: 10.1210/er.2018-00139 30124805 PMC6260247

[4] Rossi GP, Bernini G, Desideri G, Fabris B, Ferri C, Giacchetti G, et al. Renal damage in primary aldosteronism: results of the PAPY Study. Hypertension. 2006;48(2):232–238. doi: 10.1161/01.HYP.0000230444.01215.6a 16801482

[5] Monticone S, Sconfienza E, D’Ascenzo F, Buffolo F, Satoh F, Sechi LA, et al. Renal damage in primary aldosteronism: a systematic review and meta-analysis. J Hypertens. 2020;38(1):3–12. doi: 10.1097/HJH.0000000000002216 31385870

[6] Ogata H, Yamazaki Y, Tezuka Y, Gao X, Omata K, Ono Y, et al. Renal Injuries in Primary Aldosteronism: Quantitative Histopathological Analysis of 19 Patients With Primary Adosteronism. Hypertension. 2021;78(2):411–421. doi: 10.1161/HYPERTENSIONAHA.121.17436 34120452 PMC8597934

[7] Fernández-Argüeso M, Pascual-Corrales E, Bengoa Rojano N, García Cano A, Jiménez Mendiguchía L, Araujo-Castro M. Higher risk of chronic kidney disease and progressive kidney function impairment in primary aldosteronism than in essential hypertension. Case-control study. Endocrine. 2021;73(2):439–446. doi: 10.1007/s12020-021-02704-2 33797699

[8] Iwakura Y, Morimoto R, Kudo M, Ono Y, Takase K, Seiji K et al. Predictors of decreasing glomerular filtration rate and prevalence of chronic kidney disease after treatment of primary aldosteronism: renal outcome of 213 cases. J Clin Endocrinol Metab. 2014;99(5):1593–1598. doi: 10.1210/jc.2013-2180 24285678

[9] Kobayashi H, Abe M, Nakamura Y, Takahashi K, Fujita M, Takeda Y, et al. Association Between Acute Fall in Estimated Glomerular Filtration Rate After Treatment for Primary Aldosteronism and Long-Term Decline in Renal Function. Hypertension. 2019;74(3):630–638. doi: 10.1161/HYPERTENSIONAHA.119.13131 31327258

[10] Lu YC, Liu KL, Wu VC, Wang SM, Lin YH, Chueh SJ, et al. Factors associated with renal function change after unilateral adrenalectomy in patients with primary aldosteronism. Int J Urol. 2022;29(8):831–837. doi: 10.1111/iju.14905 35474521

[11] Zhu Q, Xu Q, Dou W, Zhu W, Wu J, Chen W, et al. Diffusion kurtosis imaging features of renal cell carcinoma: a preliminary study. Br J Radiol. 2021;94(1122):20201374. doi: 10.1259/bjr.20201374 33989037 PMC8173694

[12] Dai H, Zhao C, Xiong Y, He Q, Su W, Li J, et al. Evaluation of contrast-induced acute kidney injury using IVIM and DKI MRI in a rat model of diabetic nephropathy. Insights Imaging. 2022;13(1):110. Published 2022 Jun 29. doi: 10.1186/s13244-022-01249-w 35767196 PMC9243200

[13] Liang P, Yuan G, Li S, He K, Peng Y, Hu D, et al. Non-invasive evaluation of the pathological and functional characteristics of chronic kidney disease by diffusion kurtosis imaging and intravoxel incoherent motion imaging: comparison with conventional DWI. Br J Radiol (2023) doi: 10.1259/bjr.20220644 36400040 PMC10997028

[14] Rosenkrantz AB, Padhani AR, Chenevert TL, Koh DM, De Keyzer F, Taouli B, et al. Body diffusion kurtosis imaging: Basic principles, applications, and considerations for clinical practice. J Magn Reson Imaging. 2015;42(5):1190–1202. doi: 10.1002/jmri.24985 26119267

[15] Jensen JH, Helpern JA, Ramani A, Lu H, Kaczynski K. Diffusional kurtosis imaging: the quantification of non-gaussian water diffusion by means of magnetic resonance imaging. Magn Reson Med. 2005;53(6):1432–1440. doi: 10.1002/mrm.20508 15906300

[16] Raab P, Hattingen E, Franz K, Zanella FE, Lanfermann H. Cerebral gliomas: diffusional kurtosis imaging analysis of microstructural differences. Radiology. 2010;254(3):876–881. doi: 10.1148/radiol.09090819 20089718

[17] Fieremans E, Jensen JH, Helpern JA. White matter characterization with diffusional kurtosis imaging. Neuroimage. 2011;58(1):177–188. doi: 10.1016/j.neuroimage.2011.06.006 21699989 PMC3136876

[18] Pentang G, Lanzman RS, Heusch P, Müller-Lutz A, Blondin D, Antoch G, et al. Diffusion kurtosis imaging of the human kidney: a feasibility study. Magn Reson Imaging. 2014;32(5):413–420. doi: 10.1016/j.mri.2014.01.006 24582288

[19] Cao J, Luo X, Zhou Z, Duan Y, Xiao L, Sun X, et al. Comparison of diffusion-weighted imaging mono-exponential mode with diffusion kurtosis imaging for predicting pathological grades of clear cell renal cell carcinoma. Eur J Radiol. 2020;130:109195. doi: 10.1016/j.ejrad.2020.109195 32763475

[20] Ding Y, Tan Q, Mao W, Dai C, Hu X, Hou J, et al. Differentiating between malignant and benign renal tumors: do IVIM and diffusion kurtosis imaging perform better than DWI?. Eur Radiol. 2019;29(12):6930–6939. doi: 10.1007/s00330-019-06240-6 31161315

[21] Li S, He K, Yuan G, Yong X, Meng X, Feng C, et al. WHO/ISUP grade and pathological T stage of clear cell renal cell carcinoma: value of ZOOMit diffusion kurtosis imaging and chemical exchange saturation transfer imaging. Eur Radiol. 2023;33(6):4429–4439. doi: 10.1007/s00330-022-09312-2 36472697

[22] Wang B, Wang Y, Li L, Guo J, Wu PY, Zhang H, et al. Diffusion kurtosis imaging and arterial spin labeling for the noninvasive evaluation of persistent post-contrast acute kidney injury. Magn Reson Imaging. 2022;87:47–55. doi: 10.1016/j.mri.2021.12.004 34968702

[23] Li X, Liang Q, Ouchi E, Bautista M, Hu J, Zhang X. Using Multi-model Diffusion Weighted Imaging to Study Acute Kidney Injury in Patients with Acute Pancreatitis. Curr Med Imaging. 2023;19(12):1404–1414. doi: 10.2174/1573405619666230130123138 36717989

[24] Mao W, Ding Y, Ding X, Fu C, Zeng M, Zhou J. Diffusion kurtosis imaging for the assessment of renal fibrosis of chronic kidney disease: A preliminary study. Magn Reson Imaging. 2021;80:113–120. doi: 10.1016/j.mri.2021.05.002 33971241

[25] Lin J, Zhu C, Cui F, Qu H, Zhang Y, Le X, et al. Based on functional and histopathological correlations: is diffusion kurtosis imaging valuable for noninvasive assessment of renal damage in early-stage of chronic kidney disease? [published online ahead of print, 2023 Jun 16]. Int Urol Nephrol. 2023;10.1007/s11255-023-03632-y. doi: 10.1007/s11255-023-03632-y 37326823

[26] Zhou H, Zhang J, Zhang XM, Chen T, Hu J, Jing Z et al. Noninvasive evaluation of early diabetic nephropathy using diffusion kurtosis imaging: an experimental study. Eur Radiol. 2021;31(4):2281–2288. doi: 10.1007/s00330-020-07322-6 32997177

[27] Cheng ZY, Chen PK, Feng YZ, Chen XQ, Qian L, Cai XR. Preliminary Feasibility Study on Diffusion Kurtosis Imaging to Monitor the Early Functional Alternations of Kidneys in Streptozocin-Induced Diabetic Rats [published online ahead of print, 2022 Oct 13]. Acad Radiol. 2022;S1076-6332(22)00507-4. doi: 10.1016/j.acra.2022.09.016 36244869

[28] Liang P, Li S, Yuan G, He K, Li A, Hu D, et al. Noninvasive assessment of clinical and pathological characteristics of patients with IgA nephropathy by diffusion kurtosis imaging. Insights Imaging. 2022;13(1):18. Published 2022 Jan 29. doi: 10.1186/s13244-022-01158-y 35092495 PMC8800983

[29] Cao Y, Yin J, Hu M, Cui F, Qu H, Zhang Y, et al. Evaluating the renal mild tubulointerstitial damage and renal function in IgAN patients: a comparative study based on diffusion kurtosis imaging and diffusion tensor imaging. Abdom Radiol (NY). 2023;48(4):1350–1362. doi: 10.1007/s00261-023-03822-3 36749369

[30] Zhang S, Lin Y, Ge X, Liu G, Zhang J, Xu S, et al. Multiparameter diffusion-weighted imaging for characterizing pathological patterns in lupus nephritis patients: A preliminary study. J Magn Reson Imaging. 2019;50(4):1075–1084. doi: 10.1002/jmri.26657 30659687

[31] Bennett KM, Schmainda KM, Bennett RT, Rowe DB, Lu H, Hyde JS. Characterization of continuously distributed cortical water diffusion rates with a stretched-exponential model. Magn Reson Med. 2003;50(4):727–734. doi: 10.1002/mrm.10581 14523958

[32] Rogers HJ, Verhagen MV, Clark CA, Hales PW. Comparison of models of diffusion in Wilms’ tumours and normal contralateral renal tissue. MAGMA. 2021;34(2):261–271. doi: 10.1007/s10334-020-00862-4 32617696 PMC8018931

[33] Wen D, Xu C, Deng L, Yan W, Peng P, Yue X, et al. Monoexponential, biexponential, stretched-exponential and kurtosis models of diffusion-weighted imaging in kidney assessment: comparison between patients with primary aldosteronism and healthy controls. Abdom Radiol (NY). 2023;48(4):1340–1349. doi: 10.1007/s00261-023-03833-0 36745206

[34] Funder JW, Carey RM, Mantero F, Murad MH, Reincke M, Shibata H, et al. The Management of Primary Aldosteronism: Case Detection, Diagnosis, and Treatment: An Endocrine Society Clinical Practice Guideline. J Clin Endocrinol Metab. 2016;101(5):1889–1916. doi: 10.1210/jc.2015-4061 26934393

[35] Liu X, Zhou L, Peng W, Wang H, Zhang Y. Comparison of stretched-Exponential and monoexponential model diffusion-Weighted imaging in prostate cancer and normal tissues. J Magn Reson Imaging. 2015;42(4):1078–1085. doi: 10.1002/jmri.24872 25727776

[36] Kusunoki M, Kikuchi K, Togao O, Yamashita K, Momosaka D, Kikuchi Y, et al. Differentiation of high-grade from low-grade diffuse gliomas using diffusion-weighted imaging: a comparative study of mono-, bi-, and stretched-exponential diffusion models. Neuroradiology. 2020;62(7):815–823. doi: 10.1007/s00234-020-02456-2 32424712 PMC7311374

[37] Li C, Ye J, Prince M, Peng Y, Dou W, Shang S, et al. Comparing mono-exponential, bi-exponential, and stretched-exponential diffusion-weighted MR imaging for stratifying non-alcoholic fatty liver disease in a rabbit model. Eur Radiol. 2020;30(11):6022–6032. doi: 10.1007/s00330-020-07005-2 32591883

[38] Wang GZ, Guo LF, Gao GH, Li Y, Wang XZ, Yuan ZG. Magnetic Resonance Diffusion Kurtosis Imaging versus Diffusion-Weighted Imaging in Evaluating the Pathological Grade of Hepatocellular Carcinoma. Cancer Manag Res. 2020;12:5147–5158. Published 2020 Jun 29. doi: 10.2147/CMAR.S254371 32636677 PMC7334009

[39] Feng YZ, Ye YJ, Cheng ZY, Hu JJ, Zhang CB, Qian L, et al. Non-invasive assessment of early stage diabetic nephropathy by DTI and BOLD MRI. Br J Radiol. 2020;93(1105):20190562. doi: 10.1259/bjr.20190562 31603347 PMC6948087

[40] Li LP, Milani B, Pruijm M, Kohn O, Sprague S, Hack B, et al. Renal BOLD MRI in patients with chronic kidney disease: comparison of the semi-automated twelve layer concentric objects (TLCO) and manual ROI methods. MAGMA. 2020;33(1):113–120. doi: 10.1007/s10334-019-00808-5 31823276

